# Glycogen storage disease type 1a in the Ohio Amish

**DOI:** 10.1002/jmd2.12310

**Published:** 2022-06-21

**Authors:** Ethan M. Scott, Olivia K. Wenger, Elizabeth Robinson, Kristina Colling, Miraides F. Brown, Jennifer Hershberger, Kadakkal Radhakrishnan

**Affiliations:** ^1^ New Leaf Center Clinic for Special Children Ohio USA; ^2^ Department of Pediatrics Akron Children's Hospital Akron Ohio USA; ^3^ Department of Pediatric Gastroenterology, Hepatology, and Nutrition Cleveland Clinic Foundation Cleveland Ohio USA; ^4^ Akron Children's Hospital Rebecca D Considine Research Institute Akron Ohio USA

**Keywords:** gross motor delay, hepatomegaly, hypertriglyceridemia, hypoglycemia, oral aversion, quality of life

## Abstract

Glycogen storage disease type 1a (GSD1a) is an inborn error of glucose metabolism characterized by fasting hypoglycemia, hepatomegaly, and growth failure. Late complications include nephropathy and hepatic adenomas. We conducted a retrospective observational study on a cohort of Amish patients with GSD1a. A total of 15 patients cared for at a single center, with a median age of 9.9 years (range 0.25–24 years) were included. All patients shared the same founder variant in *GCPC* c.1039 C > T. The phenotype of this cohort demonstrated good metabolic control with median cohort triglyceride level slightly above normal, no need for continuous overnight feeds, and a higher quality of life compared to a previous GSD cohort. The most frequent complications were oral aversion, gross motor delay, and renal hyperfiltration. We discuss our unique care delivery at a single center that cares for Amish patients with inherited disorders.

**Synopsis**

The Ohio Amish with GSD1a have few hypoglycemic events and report a high quality of life; however, oral aversion and gross motor delay are common.

## 1 INTRODUCTION

Glycogen storage disease type Ia (GSD1a) is an autosomal recessive inborn error of glucose metabolism.^1^, ^2^, ^3^, ^4^, ^5^, ^6^, ^7^ GSD1a is caused by a deficiency of glucose‐6‐phosphatase.^1^, ^5^, ^6^, ^7^, ^8^, ^9^, ^10^ Deficiency of glucose‐6‐phoshatase prevents the final step in the gluconeogenesis and glycogenolysis pathway with excess glycogen becoming trapped in the liver and kidneys.^1^, ^4^, ^11^, ^12^ Infants with GSD1a typically present between 3 and 6 months of life with fasting hypoglycemia, hepatomegaly, growth failure, and developmental delay.^1^, ^2^, ^3^, ^6^, ^7^, ^13^, ^14^, ^15^ Laboratory derangements include: hypoglycemia, lactic acidosis, hypertriglyceridemia, and hyperuricemia.^1^, ^6^, ^7^, ^11^, ^14^, ^16^, ^17^ Complications due to poor metabolic control present in adulthood and include: hepatic adenomas with potential for dysplasia, protein losing nephropathy, short stature, and osteoporosis.^2^, ^7^, ^12^, ^16^, ^18^, ^19^, ^20^, ^21^, ^22^, ^23^, ^24^ Therapy is primarily nutritional and uses a combination of uncooked cornstarch, frequent meals, and at times continuous overnight feeds.^2^, ^3^, ^7^, ^12^, ^16^, ^22^, ^25^, ^26^

GSD1a is caused by over 100 different variants in the *G6PC* gene with a global incidence of 1:100000.^5^, ^8^, ^9^, ^27^ However, there are ethnic groups that due to a founder variant have an increased incidence.^5^, ^7^, ^8^, ^28^ The Ohio Amish with GSD1a are homozygous for the c.1039 C > T (p.Gln347*) variant in *G6PC*.^7^ The Ohio Amish have a carrier frequency of 1 in 50. (E. Baple and A. Crosby personal communication September 25, 2020). There have been case reports of possible genotype–phenotype correlations, but to date there has not been a focused analysis of the Amish variant of GSD1a.^5^, ^8^, ^9^, ^27^, ^28^ Genotype–phenotype descriptions will continue to be important to judge the efficacy of current treatment against emerging gene therapies.^2^, ^24^, ^25^, ^29^, ^30^, ^31^, ^32^

The primary objective of this study was to characterize the clinical, laboratory, growth parameters, and quality of life of a genetically homogenous group of Amish patients with GSD1a cared for at a single center.

## 2 METHODS

Clinical data was retrospectively collected from medical records and a parent questionnaire of GSD1a patients seen at New Leaf Center Clinic for Special Children from January 2013 to February 2022.

Patients were diagnosed clinically, followed by molecular confirmation or by cord blood analysis if there was a family history.

Initial record review noted age of symptom onset, age of diagnosis, presenting symptoms, and age of cornstarch introduction.

Variables at the most recent clinic visit included height (Z‐score), weight (Z‐score), BMI (Z‐score), triglycerides, bicarbonate, glucose, uric acid, GFR (calculated by Bedside Schwartz method), urine protein/creatinine ratio, vitamin D, hepatic US/MRI, and cornstarch dose.

Assessment for adequate metabolic control was adapted from the European Study on Glycogen Storage Disease 1 guidelines defined as blood glucose >63 mg/dl, uric acid <7 mg/dl, venous bicarbonate >20 mEq/L, triglycerides <370 mg/dl, and BMI within two SDs at last visit.^12^ A more stringent triglycerides limit of <370 mg/dl was used which has been shown to decrease adenoma formation and nephropathy.^16^ A patient was considered adherent to treatment if he/she had no missed appointments, hospitalizations, or episodes of symptomatic hypoglycemia (irritability, tremulousness, and seizures) in the past 12 months.^33^

Complications were assessed by organ system. Gastrointestinal complications included short stature (height Z‐score <2), oral aversion, vitamin D deficiency (< 30 ng/mL), and presence of hepatic adenomas. Renal complications included hyperfiltration (calculated GFR >140), proteinuria (>0.2 mg protein/ mg creatinine), nephrolithiasis, and hypertension. For patients <13 years, hypertension was defined as a blood pressure ≥ 95th percentile for age, sex, and height on three consecutive measurements. For patients >13 years, hypertension was defined as a systolic blood pressure > 130 and diastolic blood pressure > 80 on three consecutive measurements.^34^ Hematologic complications included anemia (hemoglobin <10 g/dl) and bleeding diathesis. Neurocognitive complications included gross motor delay, special education enrollment, and seizures.^2^

The Peds QL4.0 Generic Core Scales were used to assess quality of life in patients and their parents. To compare quality of life to a clinical cohort, the PedsQL 4.0 scores in our patients were examined against a previously reported GSD1 cohort and healthy control cohort.^35^

### 2.1 Statistical analysis

A convenience sample was used for the small population due to the rare nature of the patients included in this study.

Descriptive statistics including frequency for categorical data, and mean, median, SD for continuous data were primarily used. Student T‐test was used to compare quality of life between current and historical cohorts. Because each variable was compared twice (current vs historical; current vs healthy), alpha was adjusted using Bonferroni method, with new adjusted alpha; 0.05/2 = 0.025

Statistical analysis was completed using SAS 9.4 © (Cary, NC).

## 3 RESULTS

Fifteen GSD1a patients were enrolled. All patients were homozygous for the Amish founder variant, *G6PC* c.1039 C > T (p.Gln347*). There were eight males and seven females.

### 3.1 Diagnosis and Initial Treatment

Initial clinical findings and cornstarch dosing are in Table 1. Hypoglycemia and hepatomegaly were present in all 10 symptomatic patients. The median age of symptom onset was 0.75 months (range 0.25–12 months). Symptomatic patients were diagnosed at a median age of 11 months (range 5–25 months). Median delay in diagnosis from symptom onset was 8 months (range 1.5–24.5 months).

**TABLE 1:** Initial clinical findings and treatment of Ohio Amish GSD1a patients

Patient ID#	Gender	Age at Symptoms (months)	Age at diagnosis (months)	Delay in diagnosis (months)	Symptomatic hypoglycemia	Hepatomegaly	Failure to thrive	Age cornstarch initiated (months)	Initial Cornstarch dose in g/kg
1	M	2	17	15	+	+	+	17	n/a
2	F	3.5	5	1.5	+	−	−	6	n/a
3	F	12	20	8	−	+	+	20	0.2 q4 h
4	M	0.5	6	5.5	+	+	−	6	n/a
5	F	0.5	11	10.5	+	+	+	11	1.3 q4 h
6	M	3	11	8	+	+	+	11	1.2 q6 h
7	M	0.5	25	24.5	+	+	−	25	0.2 q3 h
8	M	1	9	8	+	+	−	10	1. 9 q4 h
9	F	n/a	0.5 (cord blood)	0	−	−	−	1	0.6 q3 h
10	F	n/a	0.5 (cord blood)	0	−	−	−	0.5	0. 3 q3 h
11	M	0.25	8	7.75	+	+	−	8	1.0 q3 h
12	M	0.5	12	11.5	+	+	+	12	1.3 q3 h
13	F	n/a	0.5 (cord blood)	0	−	−	−	2	0.6 q4 h
14	M	n/a	0.5 (cord blood)	0	−	−	−	0.5	0. 7 q3 h
15	F	n/a	0.5 (cord blood)	0	−	−	−	4.5	0.3 q8h

Five patients received a molecular diagnosis prior to symptom onset by targeted cord blood testing.

Patients clinically diagnosed initiated cornstarch at a median of 11 months (range 6–25 months) with a starting dose of 0.2–1.9 g/kg/dose. Patients diagnosed by cord blood initiated cornstarch at a median age of 2 months (range 0.5–4.5 months). The initial dose range was 0.3–0.7 g/kg/dose. No formal fasting tolerance studies were completed; however, patients did not go longer than 4 h overnight between feeds. The only reported side effect of cornstarch was loose stools, but there were no cases of discontinuation due to adverse effects, even in patients who started cornstarch prior to 6 months of age.

### 3.2 Metabolic control and dietary treatment

Most recent visit findings are in Table 2. The median age at last follow‐up was 9.92 years (range 0.25–24 years). Ten patients (67%) had good metabolic control. The median triglycerides for the cohort were 189 mg/dl (range 136–435).

**TABLE 2:** Metabolic control at last clinic visit in Ohio Amish GSD1a patients

Patient ID#	Age at last visit	Adequate metabolic control	Glucose mg/dl	Uric acid mg/dl	Bicarbonate	Triglycerides	BMI (Z‐score)	Current Cornstarch Dose in g/kg
1	24‐year 0 month	Yes	85	5.5	29.1	151	−1.48	1.8 q4 h
2	20‐year 0 month	Yes	90	6.3	31.2	145	0.15	1.2 q4 h
3	17‐year 0 month	Yes	99	6	25.7	157	1.65	1.5 q6 h
4	15‐year 10 month	No	53	5.2	23.4	136	0.31	2.1 q5 h
5	14‐year 11 month	Yes	86	7.3	22	241	0.41	1.9 q6 h
6	11‐year 5 month	Yes	90	5.1	26.2	170	1.08	1.2 q5 h
7	10‐year 5 month	Yes	102	4.9	24.4	189	0.74	1.9 q5 h
8	9‐year 11 month	Yes	66	4.7	24	226	0.39	2.6 q5 h
9	6‐year 11 month	No	90	7.4	23	307	2.05	1.0 q5 h
10	5‐year 7 month	Yes	132	4.6	22.1	142	0.50	1.5 q5 h
11	4‐year 7 month	Yes	86	3.8	25.1	78	1.18	1.6 q3.5 h
12	2‐year 8 month	No	61	5.9	18.4	196	0.55	1.0 q3 h
13	1 year 1 month	Yes	97	5.9	26.3	256	1.04	1.7 q4 h
14	1 year 2 month	No	65	7.2	26.8	255	2.70	0. 7 q3 h
15	0‐year 3 month	No	61	4.5	21.2	435	0.99	0.3 q3 h

*Note*: Areas that are shaded show where adequate metabolic control was not met.

The mean cornstarch dose was 1.54 g/kg/dose every 3–6 h (range 0.7–2.6 g/kg/dose). No patients required overnight gastrostomy feeds.

### 3.3 Adherence to treatment

The 13 patients (87%) had good adherence to treatment. Patients #4 and 12 did not meet criteria for good adherence due to symptomatic hypoglycemia treated at home.

### 3.4 Complications and comorbidities

Organ system complications are in Table 3. Only patient #1 had a hepatic adenoma. Multiple adenomas were identified on MRI at 20 years old. All but one of these lesions had regressed on last MRI.

**TABLE 3:** Complications by organ system in Ohio Amish GSD1a patients

Patient ID#	Age at last visit	Short stature (height z‐score <2)	Oral Aversion	Vit. D deficiency	Hepatic adenoma	Hyperfiltration	Proteinuria	Nephrolithiasis	Hypertension
1	24 year 0 month	−	−	−	+	−	−	+	−
2	20 year 0 month	−	−	−	−	−	−	−	−
3	17 year 0 month	−	+	−	−	−	−	−	−
4	15 year 10 month	−	+	−	−	−	−	−	−
5	14 year 11 month	−	−	−	−	+	−	−	−
6	11 year 5 month	+	−	+	−	+	+	−	−
7	10 year 5 month	−	−	−	−	+	−	−	−
8	9 year 11 month	−	+	−	−	+	−	−	+
9	6 year 11 month	−	+	−	−	+	+	−	+
10	5 year 7 month	−	+	−	−	+	+	−	+
11	4 year 7 month	−	−	−	−	+	+	−	−
12	2 year 8 month	−	−	+	−	−	n/a	−	+
13	1 year 1 month	−	+	−	−	−	n/a	−	−
14	1 year 2 month	−	+	+	−	+	n/a	−	−
15	0 year 3 month	−	−	−	−	−	n/a	−	−
Total		1/15	8/15	3/15	1/15	8/15	4/11	1/15	4/15

Patient ID#	Age at last visit	Anemia	Bleeding diathesis	Gross motor delay	Cognitive impairment	History of seizures	Symptomatic Hypoglycemic Episodes 12 months	Metabolic hospitalizations in past 12 months	Zero Missed appointments
1	24 year 0 month	−	−	+	−	−	−	−	+
2	20 year 0 month	−	−	−	−	−	−	−	+
3	17 year 0 month	−	−	−	−	−	−	−	+
4	15 year 10 month	−	−	+	−	−	+	−	+
5	14 year 11 month	−	−	−	−	−	−	−	+
6	11 year 5 month	−	−	+	+	−	−	−	+
7	10 year 5 month	−	−	−	−	−	−	−	+
8	9 year 11 month	−	−	+	−	−	−	−	+
9	6 year 11 month	−	−	+	−	−	−	−	+
10	5 year 7 month	−	−	+	−	+ *(non‐GSD related)*	−	−	+
11	4 year 7 month	−	−	−	−	+	−	−	+
12	2 year 8 month	−	−	+	−	−	+	−	+
13	1 year 1 month	−	−	−	−	−	−	−	+
14	1 year 2 month	−	−	+	−	+	−	−	+
15	0 year 3 month	−	−	−	−	−	−	−	+
Total		0/15	0/15	8/15	1/15	3/15	2/15	0/15	15/15

Three patients had seizures (20%), including a sibling pair, none required antiepileptic therapy.

### 3.5 Quality of life

Quality of life measured by the PedsQL 4.0 is in Supplementary Table 1. The current GSD cohort by patient and parent report showed better physical, psychosocial, social, and total quality of life compared to the historical GSD cohort. Both patients and parents of our GSD cohort reported a higher quality of life in social functioning compared to the historical healthy cohort.

## 4 DISCUSSION

The present observational, descriptive study investigated the clinical, biochemical, and quality of life of a genetically homogenous cohort of GSD1a patients from the Ohio Amish.

Diagnostic delay was common in our cohort with a median delay of 8 months for clinically diagnosed patients from symptom onset to diagnosis, similar to previous cohorts.^33^ Families with an affected sibling opted to have subsequent siblings tested by cord blood analysis. Cord blood testing resulted in 33% of our cohort receiving a molecular diagnosis prior to symptoms by 2 weeks of age. However, a pre‐symptomatic diagnosis did not necessarily prevent complications. Patients diagnosed via cord blood had higher rates of oral aversion and similar rates of gross motor delay compared to clinically diagnosed patients. However, it appears that early diagnosis may need to be paired with early intervention services to be impactful.

Strict genotype–phenotype associations have been difficult to establish with GSD1a.^9^ However, the *G6PC* c.1039 C > T variant in this cohort appears milder biochemically and clinically. The median triglyceride level in our cohort was in the borderline elevated range and all but one patient attained a triglyceride level < 370 which has been associated with decreased adenoma formation, osteoporosis, and nephropathy.^16^ However, this may also be reflective of better acceptance and compliance of treatment in our cohort with improved access to local care. Only 1 patient (7%) had short stature which is less than other recently reported cohorts with an incidence between 20% and 40%.^33^, ^36^, ^37^ The normal height in the majority of patients likely reflects the good metabolic control, which has previously been shown to improve growth.^36^ Further no patients required continuous overnight feeds, there were no hospitalizations in the past 12 months due to a hypoglycemic complication, and no patients required liver transplant.

GSD associated feeding difficulties are being increasingly recognized.^38^, ^39^, ^40^ Oral aversion was reported in 47% of our patients, which is similar to findings from Martinez et al who found that 30% of patients with GSD had oral aversion, compared to of 0.25% in the general population.^39^, ^41^ All patients who started cornstarch within 2 months of birth, who had been diagnosed by cord blood developed oral aversion. Our cohort had high rates of oral aversion despite no patients requiring an alternative feeding route which is a contributing factor to oral aversion.^12^, ^40^, ^42^ We suspect that early cornstarch introduction led to near constant satiety, while beneficial for metabolic control was deleterious to the acquisition of oral feeding skills. None of the patients in this cohort demonstrated intolerance to corn starch. We would recommend delaying cornstarch introduction at least until whole food introduction at approximately 6 months and early involvement of a speech therapist at any sign of oral aversion.

Hepatic complications were rare in our cohort. Only one patient was affected by hepatic adenomas (multiple identified on MRI). This likely reflects the young median age of our cohort, but potentially also the metabolic control. The adenoma in the one affected patient had shown regression with good metabolic control similar to previous reports.^33^, ^43^

Consistent with previous reports hyperfiltration was present, even in our youngest patients.^44^, ^45^ Progression of renal disease to overt nephropathy with proteinuria and decreasing GFR is more common in adulthood and our cohort is too young to capture these changes and draw definitive conclusions on the renal phenotype.^16^, ^46^

Children with GSD, similar to those with other chronic conditions, are at increased risk for impaired quality of life.^35^, ^47^ Both our patients with GSD and their parents reported a higher quality of life than a previously reported cohort with GSD. Our patients reported a similar quality of life to the general population. We suspect this is due to a milder variant which requires fewer interventions such as NG or G‐tube feeds and low rates of hospitalization. We hypothesize that, in addition to a milder variant, the care delivery (described below) may aid in their improved quality of life.

The primary limitation of this study is that it is a small, single‐center, retrospective study. Additionally, our center has a young cohort, many of which are too young to be at risk for several of the late complications of GSD1a. Finally, a current “healthy cohort” from within the Amish community could have provided a more meaningful exploration of the differences in quality of life.

### 4.1 Care delivery

New Leaf Center serves as a medical home as defined by the American Academy of Pediatrics, by providing cost‐effective genetic diagnostics and complex care, as well as primary care to the Amish and Mennonite (Plain) communities of Ohio who suffer from rare disorders such as GSD.^48^ This model of care has been successfully pioneered by institutions such as the Clinic for Special Children in Lancaster, PA.^49^

Care for inherited disorders can be disjointed, expensive, and time intensive requiring multiple trips to subspecialty appointments. At New Leaf we host our GSD patients for routine visits involving their primary care physician (PCP), hepatologist, nurse practitioner, and dietician in one room with the patient and family. This allows for seamless communication between the providers and families. Additionally, New Leaf is located within the community which it serves, decreasing expense, and travel time. Finally, this model allows GSD patients to be seen locally for urgent visits. Their PCP develops plans in conjunction with the offsite GSD care team allowing for more rapid assessment and treatment.

Our center strives to provide early diagnoses to prevent prolonged and expensive diagnostic odysseys. It is concerning, that even though New Leaf is embedded within the Amish community and there is a known GSD founder variant that a diagnostic delay of several months existed in many patients. This highlights the need for ongoing community education about common conditions seen within our population. It will also be prudent to explore new avenues for earlier diagnosis. GSD founder variants can now be identified using dried blood spots as well as on commercial panel based tests that target inherited diseases within the Plain communities.^50^, ^51^, ^52^ These strategies could be considered within the genetically homogenous Amish GSD population as a method for precision newborn screening.

## 5 CONCLUSION

Amish populations originating in Ohio and throughout north America's Midwest have a high incidence of GSD1a. Early diagnosis is possible, but care should be taken to be proactive to address frequent complications of oral aversion and gross motor delay. This variant appears comparatively mild as most patients demonstrated good metabolic control, did not require continuous overnight feeds, had few hypoglycemic complications, and reported a high quality of life. This may also be reflective of better acceptance and compliance of the patients to the treatment provided. Despite the relatively milder presentation, patients will need to be followed longitudinally to evaluate for potential long term complications. Our model of care could be replicated in other genetically isolated populations who are at risk for complex disorders.

## FUNDING INFORMATION

New Leaf Center Clinic for Special Children self‐funded this project.

## CONFLICT OF INTEREST

Ethan M. Scott, Olivia K. Wenger, Jennifer Hershberger, Elizabeth Robinson, Kristina Colling, Miraides F. Brown, and Kadakkal Radhakrishnan declare that they have no conflict of interest.

## INFORMED CONSENT

All procedures followed were in accordance with the ethical standards of the responsible committee on human experimentation (institutional and national) and with the Helsinki Declaration of 1975, as revised in 2000. Informed consent was obtained from all patients for being included in the study.

## ANIMAL RIGHTS

This article does not contain any studies with animal subjects performed by any of the authors.

## Supporting information


**Supplementary Table 1** Quality of Life Analysis of Ohio Amish GSD cohort compared with historic GSD cohort and historic healthy cohort

## Data Availability

The data that support the findings of this study are available from the corresponding author, Ethan M. Scott upon reasonable request.
