# Supplementary material for: Glycogen storage disease type 1a in the Ohio Amish
Source: JIMD Rep. 2022 Jun 21;63(5):453–61. doi: 10.1002/jmd2.12310 (PMC9458600; doi:10.1002/jmd2.12310)
Supplement: Supplementary file 1 — Supplementary Table 1 Quality of Life Analysis of Ohio Amish GSD cohort compared with historic GSD cohort and historic healthy cohort [file JMD2-63-453-s001.docx]

|  |  | Ohio Amish GSD Cohort (*n*=15) | | |  | Historical GSD Cohort (*n*=31) | |  | Historical Healthy Cohort (n=5480) | |
| --- | --- | --- | --- | --- | --- | --- | --- | --- | --- | --- |
| PATIENT |  | n | Mean | Standard Deviation |  | Mean | Standard Deviation |  | Mean | Standard Deviation |
|  | Total score | 10 | 85.17 | 8.84 |  | 71.65 | 9.66 |  | 83.84 | 12.65 |
|  | Physical functioning | 10 | 88.26 | 10.03 |  | 75.63 | 14.15 |  | 87.53 | 13.5 |
|  | Psychosocial health | 10 | 84.83 | 8.98 |  | 70.33 | 10.73 |  | 81.87 | 14.09 |
|  | Emotional functioning | 10 | 78.88 | 15.10 |  | 68.2 | 16.32 |  | 79.33 | 18.15 |
|  | Social functioning | 10 | 92.50 | 7.17 |  | 71.4 | 15.78 |  | 85.15 | 16.76 |
|  | School functioning | 7 | 76.43 | 13.14 |  | 71.4 | 12.46 |  | 81.12 | 16.45 |
|  |  |  |  |  |  |  |  |  |  |  |
| PARENT |  |  |  |  |  |  |  |  | (n=9430) |  |
|  | Total score | 14 | 88.6 | 10.95 |  | 74.88 | 15.64 |  | 82.7 | 15.4 |
|  | Physical functioning | 15 | 90.5 | 12.35 |  | 76.45 | 19.63 |  | 84.48 | 19.51 |
|  | Psychosocial health | 14 | 86.69 | 11.26 |  | 74.36 | 15.07 |  | 81.65 | 15.22 |
|  | Emotional functioning | 15 | 85.29 | 17.63 |  | 76.93 | 19.24 |  | 81.31 | 16.5 |
|  | Social functioning | 14 | 92.86 | 9.75 |  | 74.23 | 20.33 |  | 83.7 | 19.43 |
|  | School functioning | 10 | 82.5 | 15.86 |  | 71.92 | 18.28 |  | 78.83 | 19.59 |

**Supplementary Table 1.** Quality of Life Analysis of Ohio Amish GSD cohort compared with historic GSD cohort and historic healthy cohort
